# Portable microfluidic titration using a cross-shaped microfluidic device and smartphone camera for on-site quantitative analysis

**DOI:** 10.1007/s44211-026-00902-4

**Published:** 2026-05-12

**Authors:** Daina Numao, Nobuo Uehara, Arinori Inagawa

**Affiliations:** School of Engineering, Utsunomiya University, 7-1-2, Yoto, , Utsunomiya, Tochigi 321-8585 Japan

**Keywords:** Microfluidics, Aqueous biphasic interface, Titration, Smartphone, Image analysis

## Abstract

A portable microfluidic titration system using a smartphone camera was developed as a detector for onsite quantitative analysis. Two types of cross-shaped microfluidic devices with different channel geometries were designed and evaluated to improve the visual detectability of the equivalence point under limited spatial resolution. An expanding-channel device was found to promote molecular diffusion downstream, resulting in enhanced visibility of the equivalence point compared to a constant-width device. Using the optimized device, a strong acid–strong base system was subjected to acid–base titration using bromothymol blue as the indicator. The color images captured by the smartphone camera were analyzed by extracting the red channel intensity profiles and used to determine the distance corresponding to the equivalence point. A linear relationship was obtained between the measured distance and hydrochloric acid concentration in the range of 25–150 mM. The applicability of the proposed system was demonstrated by analyzing a real hot-spring water sample. The determined acid concentration was in good agreement with that obtained by conventional volumetric titration, with no statistically significant difference at the 95% confidence level. These results indicate that the proposed portable microfluidic titration system enables reliable quantitative analysis without the use of microscopes or specialized optical instruments, highlighting its potential for onsite analytical applications.

**Graphical abstract:**

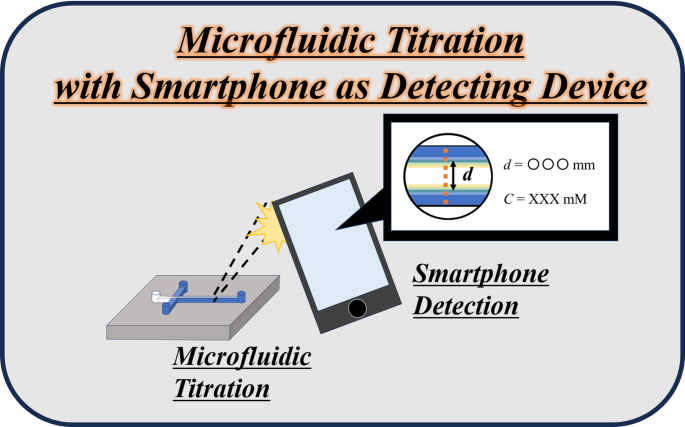

**Supplementary Information:**

The online version contains supplementary material available at 10.1007/s44211-026-00902-4.

## Introduction

In recent years, there has been a growing demand for on-site analytical methods in response to the increasing need for environmental monitoring, clinical diagnostics, and food safety assessments. On-site analysis enables the completion of sampling, pretreatment, and detection, directly at the measurement site, offering significant advantages, such as rapid decision-making, improved operational efficiency, and minimization of sample degradation or contamination during transportation.

To meet these demands, various portable analytical platforms have been developed and applied in field analyses. In the environmental field, compact gas chromatography-mass spectrometry (GC–MS) systems and portable optical instruments have been utilized for on-site measurements [1]. In the medical field, in the quantification of cardiac biomarkers, point-of-care testing (POCT) devices have been studied extensively, including portable platforms for the rapid detection of DNA [2], pathogens, and smartphone-integrated microfluidic systems [3]. These advances demonstrate the feasibility and effectiveness of portable analytical technologies in both environmental and clinical applications.

In particular, the on-site environmental analysis of real samples, such as natural waters and soils, has attracted considerable attention. Yu et al. developed a flow injection analysis system combined with chemiluminescence detection (FIA–CL) for real-time measurements of hydroxyl radicals in lake water and ambient air [4]. As conventional detection of hydroxyl radicals is often hindered by matrix interference from environmental samples, the FIA–CL approach enables sensitive and portable on-site analysis by suppressing such interference. In addition, Barzallo et al. proposed a digital image processing-based portable and multifunctional analytical system, in which colorimetric reactions are recorded using a camera and quantitatively analyzed to enable the on-site determination of analytes in water and food samples [5].

Recent advances in microfluidic technology have accelerated the development of compact and high-performance analytical devices. Microfluidic systems allow precise control of reaction conditions, reduce sample consumption, and integrate multistep processes on a single chip. The use of smartphones as detectors has emerged as a promising approach for realizing low-cost and portable analytical platforms. Temiz et al. developed a smartphone-based microfluidic flow measurement system capable of real-time analysis of sub-nanoliter flow rates [6], whereas Xia et al. demonstrated highly sensitive virus detection by analyzing colorimetric reactions induced on a microfluidic chip using a smartphone camera [7]. These studies indicate that smartphones can serve as versatile and powerful detectors for onsite microfluidic analysis without the need for specialized optical instruments.

Despite these advances, the application of titration-based analytical methods for on-site microfluidic analyses remains limited. Titration is one of the most fundamental and versatile techniques in analytical chemistry and is widely used for the quantitative determination of acids, bases, and metal ions. However, conventional titration methods typically require bulky equipment, skilled operators, and laboratory-based optical instruments, which hinder their direct application to on-site analysis. Although several microfluidic titration systems have been reported, many rely on microscopes or specialized detection setups, which limits their portability and practicality in field environments [8].

We have previously reported a microfluidic titration method based on accelerated interfacial diffusion in laminar biphasic flows [9]. In this approach, an acidic sample stream is sandwiched between titrant streams containing pH indicators, allowing protons and hydroxide ions to diffuse across the interface according to Fick’s law. Two equivalent points are formed perpendicular to the flow direction and can be identified by image-based analysis. The distance between these points, defined as *d*, correlates with the initial acid concentration and serves as a quantitative parameter for analysis. Higher concentrations result in larger *d*, whereas lower concentrations produce smaller values. This method enables simple and rapid concentration estimation without complex instrumentation and shows promise for portable and real-sample analyses. In this study, we aimed to extend microchemical titration methods to on-site analysis by developing a compact and simple measurement system using a smartphone camera as a detector. By optimizing the microchannel design and measurement conditions according to the reaction characteristics, we demonstrated that quantitative analysis of the acid concentration based on acid–base titration can be achieved without the use of a microscope. The proposed approach provides a practical and versatile platform for on-site microfluidic titration and contributes to the development of portable and user-friendly analytical systems.

## Experimental

### Apparatus

The microfluidic devices were designed using open-source computer-aided design (CAD) software (FreeCAD) and processed using slicing software (CHITUBOX). The devices were fabricated using a stereolithography-based three-dimensional (3D) printer (ELEGOO Mars Pro UV, ELEGOO) and postcured in an ultraviolet (UV)-curing box (ELEGOO Mercury). Surface modification was performed using a corona treatment system (BD-20AC, Electrotechnical Products). The glass substrates (microscope glass slides) were purchased from Matsunami Glass Inc., Ltd. The sample solutions were introduced into the microfluidic devices using gastight syringes (2.5 mL, Hamilton), laboratory tubing (TYGON LMT-55), and syringe pumps (MSPE-3, AS ONE). Color images were captured using a smartphone (iPhone 11 Pro; Apple Inc.). Image analysis and extraction of RGB values were performed using the ImageJ software developed by the National Institutes of Health (NIH, Bethesda, USA). Fluorescent images were obtained using a confocal laser scanning microscope (FLUOVIEW FV1000, Olympus).

### Reagents

The reagents comprising hydrochloric acid, sodium hydroxide, acetic acid, sodium dihydrogen phosphate, disodium hydrogen phosphate, and ethanol were purchased from Kanto Chemical Co., Inc., and used as received (special grade). Bromothymol blue (BTB) and sodium fluorescein were obtained from FUJIFILM Wako Pure Chemical Corporation. The ultrapure water used for the preparation of the aqueous solutions was produced by purifying tap water using an Elix Advantage 5 system (Millipore), followed by further purification using a Synergy UV system (Merck). The polydimethylsiloxane (PDMS) elastomer and curing agent were obtained from Dow Toray Co., Ltd.

### Fabrication of microfluidic devices

The microfluidic devices used in this study were fabricated following the procedure described previously [9], involving a 3D model design, stereolithographic 3D printing, surface treatment, and bonding to glass substrates.

**Fig. 1:**
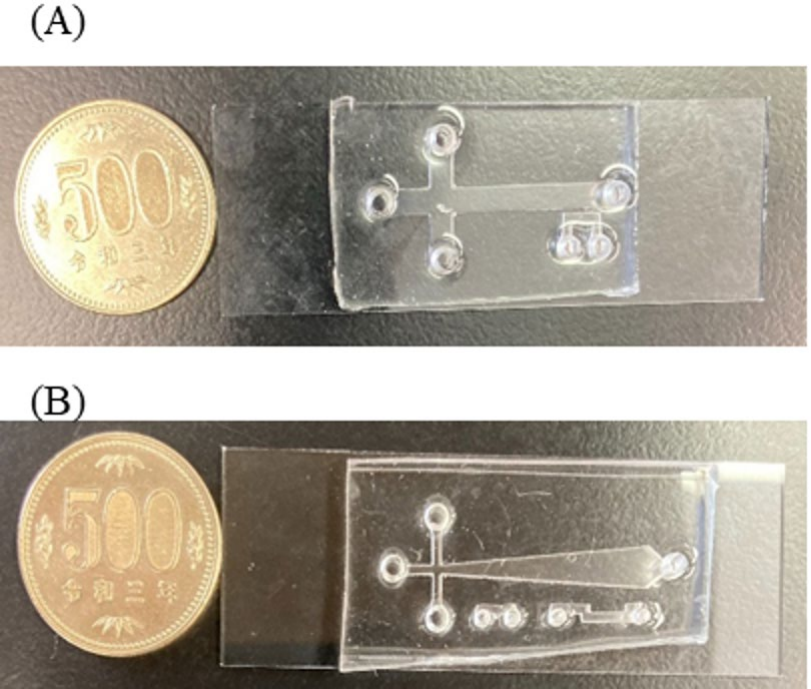
Cross-shaped microfluidic devices fabricated in this study. **A** Device with a uniform channel width of 3 mm throughout the entire channel network. **B** Device with a channel width of 1 mm at the junction, continuously expanding downstream to a maximum width of 5 mm

**Fig. 2:**
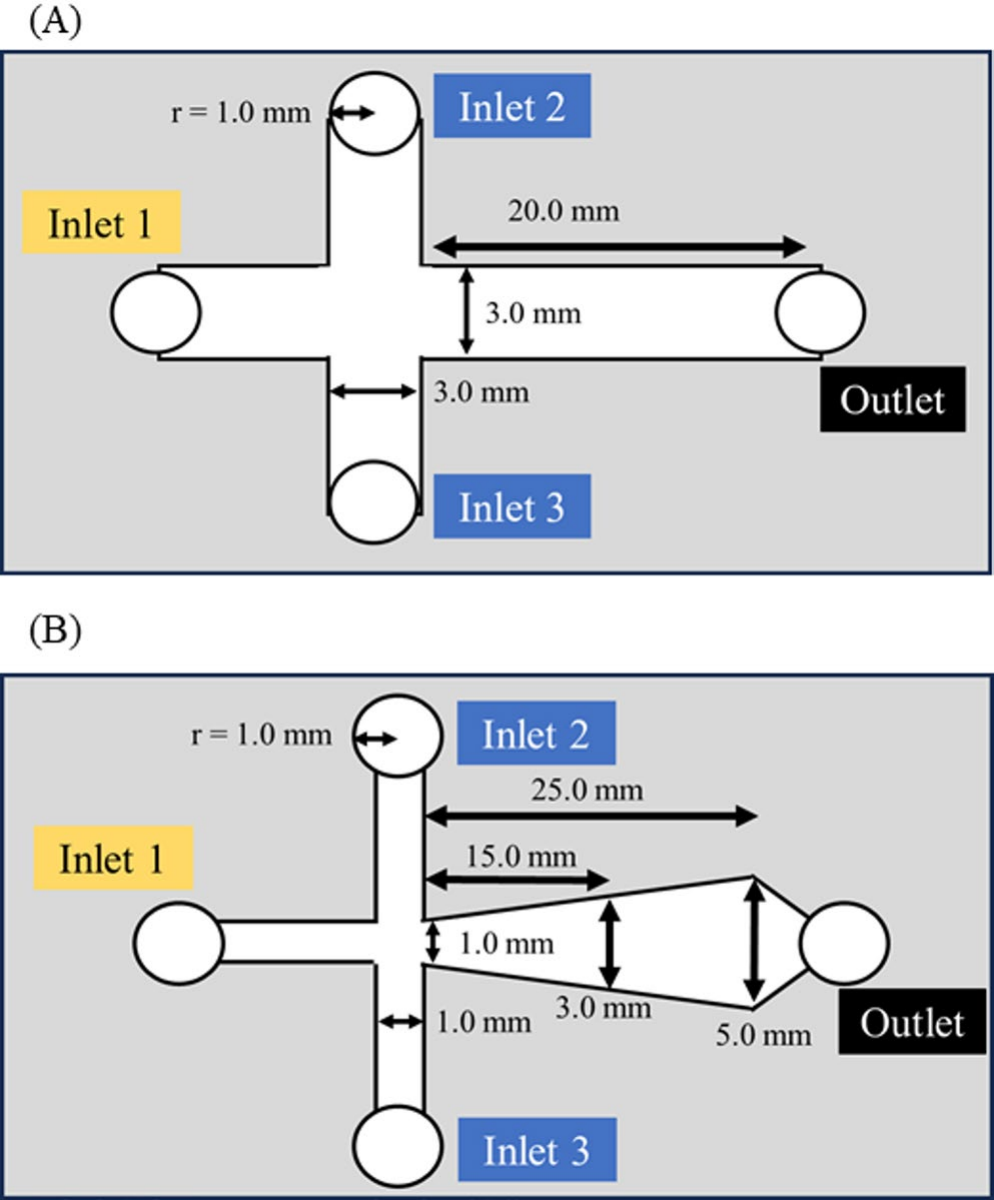
Schematic diagram of the cross-shaped microfluidic devices fabricated in this study. **A** Device with a uniform channel width of 3 mm throughout the entire channel network. **B** Device with a channel width of 1 mm at the junction, continuously expanding downstream to a maximum width of 5 mm

Two types of cross-shaped microfluidic devices with different channel geometries were fabricated. Photographs of the fabricated devices are shown in Fig. 1A and B, and schematic illustrations are shown in Fig. 2A and B. The device shown in Fig. 1A has a uniform channel width of 3 mm throughout the channel network. In contrast, the device in Fig. 1B has a channel width of 1 mm at the junction, which continuously expands downstream to a maximum width of 5 mm. The channel height was fixed at 1 mm for both devices.

### Operation of the microfluidic device

**Fig. 3:**
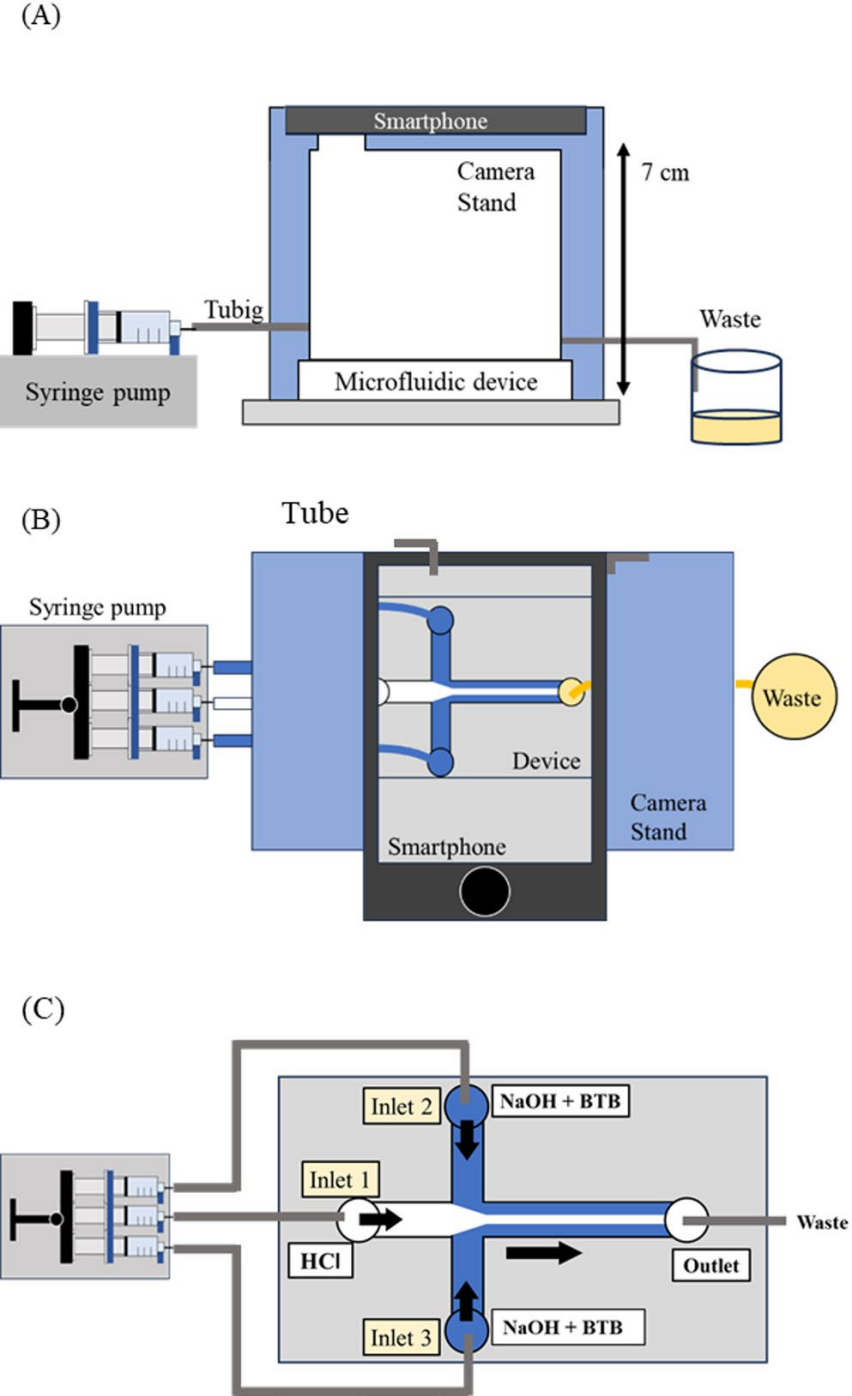
Schematic illustration of the experimental setup used in this study. **A** Side view of the portable measurement setup, in which a smartphone is mounted on a 3D-printed holder at a height of 7 cm above the microfluidic device; **B** Top view of the setup, showing the layout of the cross-shaped microfluidic device and inlet configuration. **C** Tube connection to the microfluidic device

A schematic of the experimental setup is shown in Fig. 3. The microfluidic device was operated according to a previously reported procedure [9–11]. Hydrochloric acid solutions and hot-spring water samples collected from the Tamagawa Hot Spring (Akita, Japan) were used as acidic samples. Calibration curves were constructed using hydrochloric acid solutions of known concentrations and NaOH solutions as titrants.

Unlike the previous experimental setup, no microscopes or complementary metal-oxide semiconductor (CMOS) cameras were used in this study. Instead, color changes within the microchannel were recorded using a smartphone camera. A custom-made holder fabricated by 3D printing was used to fix the smartphone 7 cm above the microfluidic device during image acquisition. The side and top views are illustrated in Fig. 3A and B, respectively. The tube connection schematically illustrated in Fig. 3C. To make the smartphone holder, we used a non-transparent gray-colored resin so that the effect of the stray light is efficiently eliminated. The photograph of the holder is shown in Fig. S1. The smartphone flash was not used in the present experiments. Image acquisition was performed under constant indoor laboratory lighting conditions. The 3D-printed holder was designed to reduce stray light rather than to provide a completely light-tight environment. The titrant solution consisted of 25 mM sodium hydroxide containing bromothymol blue at a final concentration of 8.0 × 10⁻^5^ M. Acidic samples were introduced from inlet 1, whereas the titrant solutions were introduced from inlets 2 and 3 using syringe pumps. The volumetric flow rate was set to 0.125 mL min⁻^1^, and the detection position was fixed at 1.5 cm downstream from the junction.

### Selection of RGB channel

The equivalence point within the microchannel was determined using the previously described procedure [10]. Color images captured by the smartphone camera were analyzed using the ImageJ software. The red channel was extracted from the RGB image, and red intensity values were used to generate intensity profiles perpendicular to the flow direction.

### Criteria for distance *d* measurement

**Fig. 4:**
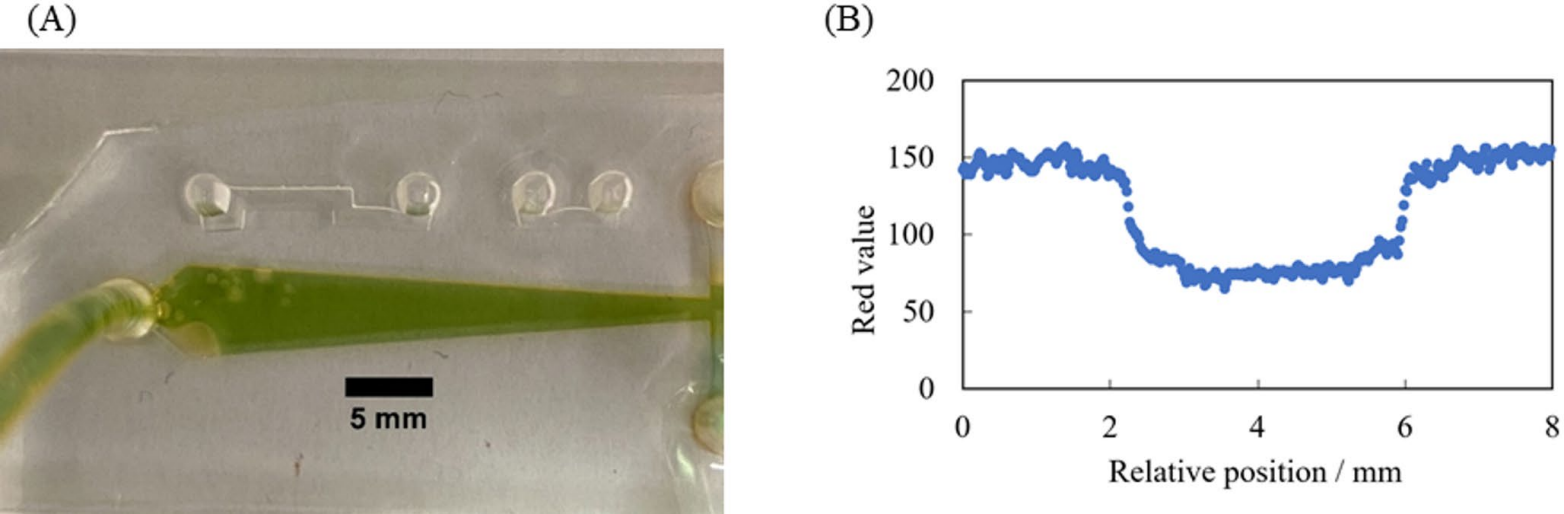
Results obtained when a pH 7.008 phosphate buffer solution was introduced into the portable microfluidic device. **A** Color image captured using a smartphone camera. **B** Red value profile extracted from the red channel image of (A) along the direction perpendicular to the flow direction

The criteria for measuring distance *d* were defined according to a previously reported method [10]. In this analysis, the reference red value corresponding to the equivalence point was defined as 77.84. This value was determined from an image obtained when a phosphate buffer solution (pH 7.008) was introduced into the portable microfluidic device, as shown in Fig. 4A. To correct for variations in image brightness between measurements, normalization was performed using a reference red value of 145.09, as shown in Fig. 4B. All titration images were normalized based on this reference prior to distance analysis. In detail, the RGB values of the white region adjacent to the microchannel were measured in each image, and the red value was used as the reference. The red values in the region of interest (ROI) within the microchannel were then normalized by correcting the difference between the measured reference value and 145.09. This normalization procedure minimizes variations in image brightness caused by ambient lighting conditions.

### Calibration curves

Calibration curves were constructed by calculating the distance *d* for hydrochloric acid solutions of known concentrations. For real-sample analysis, hot spring water collected from the Tamagawa Hot Spring was subjected to titration, and the corresponding *d* values were applied to the calibration curve to determine the acid concentration in the sample.

## Results and discussion

### Design of a portable-oriented microfluidic device and evaluation of diffusion behavior

When using a smartphone camera for image acquisition, the spatial resolution is inherently limited compared with microscopic observations. Therefore, a device design that enhances the visual distinguishability of equivalence points is essential. In microfluidic systems, channel width and cross-sectional geometry strongly influence the flow velocity distribution and mass transport via diffusion [12, 13].

Based on this consideration, two types of cross-shaped microfluidic devices were designed, as shown in Fig. 1A and B. The device shown in Fig. 1A has a constant channel width throughout the etwork and is hereafter referred to as a *constant-width device*. In contrast, the device shown in Fig. 1B has a narrow junction region followed by a continuously expanding channel downstream and is referred to as an *expanding-channel device*. In an expanding-channel structure operating at a constant volumetric flow rate, the average flow velocity decreases downstream as the channel width increases, resulting in an increased fluid residence time. The numerical simulations presented in the previous section demonstrate that a reduced flow velocity enhances the contribution of diffusion, suggesting that mass transport by diffusion is promoted in the expanding-channel device.

**Fig. 5:**
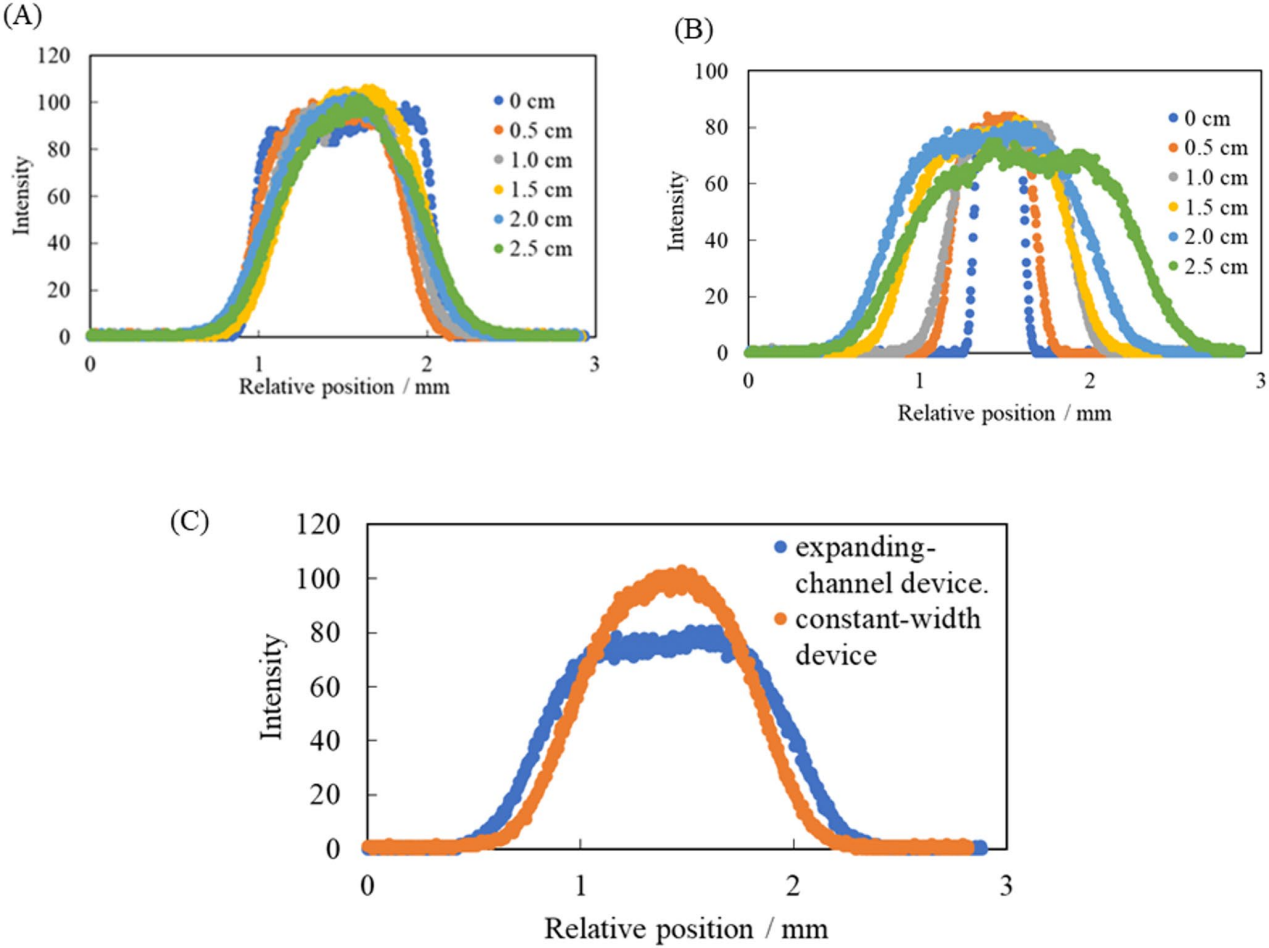
Fluorescence images and diffusion behavior of fluorescein in microfluidic devices. **A** Fluorescence images obtained in the *constant-width device* at downstream positions of 0.5 cm intervals from the junction. **B** Fluorescence images obtained in the *expanding-channel device* at downstream positions of 0.5 cm intervals from the junction. **C** Comparison of fluorescence intensity profiles at the downstream position of 1.5 cm from the junction, where the channel width is 3 mm for both devices

Diffusion experiments were conducted using fluorescein and ultrapure water in both devices to experimentally evaluate the effect of channel geometry on the diffusion behavior. A 100 μM fluorescein aqueous solution was introduced from inlet 1, and ultrapure water was introduced from inlets 2 and 3. Fluorescence images were acquired downstream at positions of 0.5 cm intervals from the junction. Fluorescence intensity profiles were extracted from the acquired images using ImageJ software, and the diffusion behavior at each downstream position was evaluated. The results are shown in Fig. 5A and B.

For a direct comparison between the two devices, the fluorescence intensity profiles at downstream positions of 1.5 cm from the junction were examined, where the channel width was 3 mm for both devices. The corresponding profiles are presented in Fig. 5C. The expanding-channel device exhibited a broader fluorescence distribution than the constant-width device, indicating enhanced diffusion of fluorescein. These results demonstrate that diffusion behavior within a microchannel can be effectively controlled by modifying the channel geometry.

Based on these findings, an expanding-channel device was selected for subsequent acid–base titration experiments to improve the visual detectability of the equivalence point under smartphone-based imaging conditions.

### Acid–base titration of a strong acid–strong base system using the portable microfluidic device

Calibration experiments were conducted using hydrochloric acid solutions of concentrations 25, 50, 75, 100, 125, and 150 mM as acidic samples. When employing the expanding-channel device, the volumetric flow rate used in the constant-width device (0.05 mL min⁻^1^ at a channel width of 1 mm) resulted in excessive diffusion due to a decrease in average flow velocity caused by the increased channel cross-sectional area. To maintain an appropriate balance between convection and diffusion, the flow rate was increased to 0.125 mL min⁻^1^ in this study. A 25 mM sodium hydroxide solution containing bromothymol blue was used as the titrant.

**Fig. 6:**
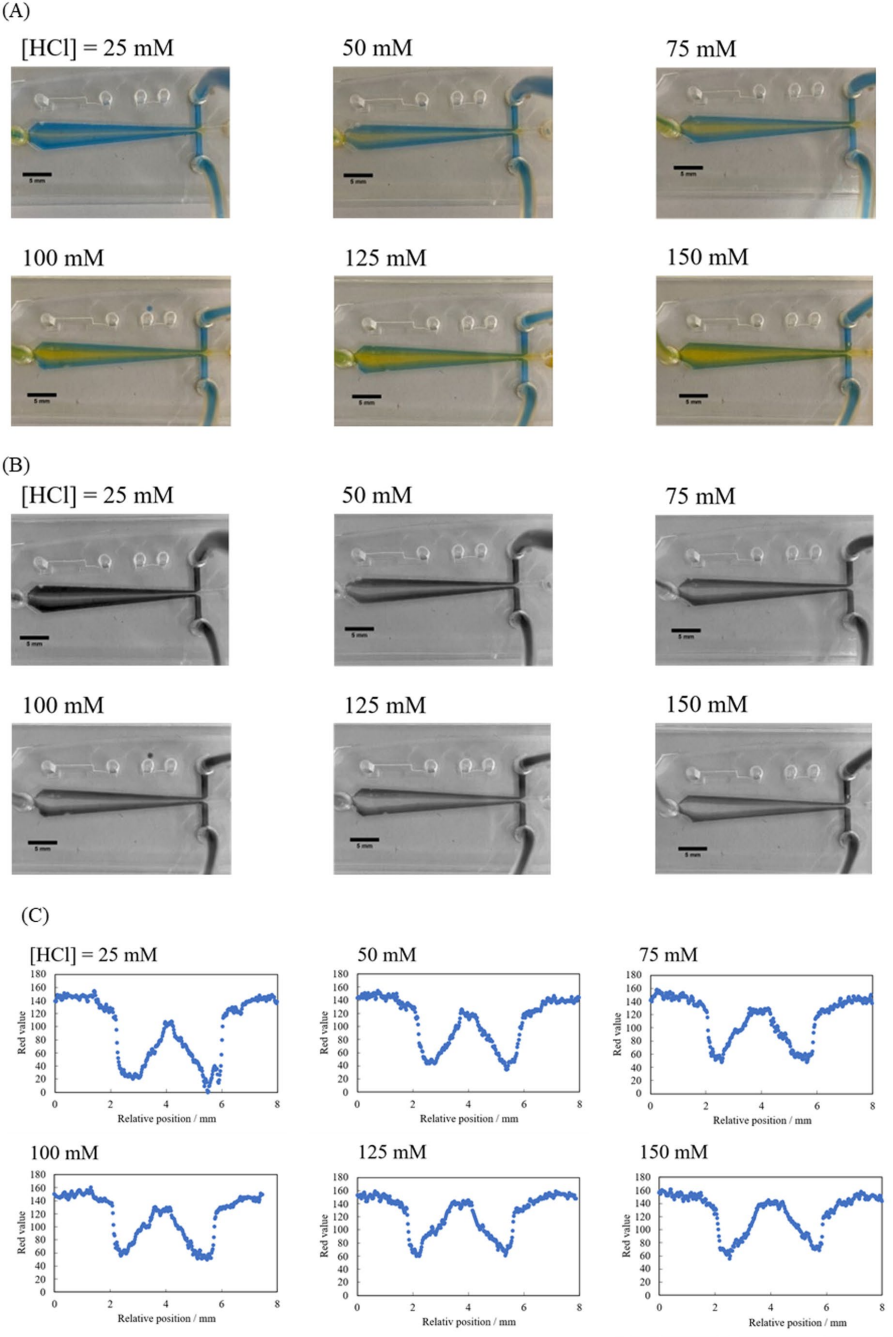
Representative results obtained during acid–base titration using a portable microfluidic titration device recorded with a smartphone camera. Hydrochloric acid solutions with concentrations of 25, 50, 75, 100, 125, and 150 mM were titrated using a 25 mM sodium hydroxide solution as the titrant. **A** Original color image acquired during titration. **B** Red channel image extracted from the smartphone-captured image shown in Fig. 6(A) using ImageJ software. **C** Red value profile of the extracted red channel along the direction perpendicular to the flow

A representative color image acquired during titration using a smartphone camera is shown in Fig. 6A. As the hydrochloric acid concentration increases, the yellow and green regions expand laterally across the channel. This observation indicates that expanding the channel geometry enhances the visual clarity of the changes at distance *d*. Notably, the color distributions corresponding to different acid concentrations are distinguishable by the naked eye, demonstrating that the device provides sufficient visibility for smartphone-based distance measurements.

**Fig. 7:**
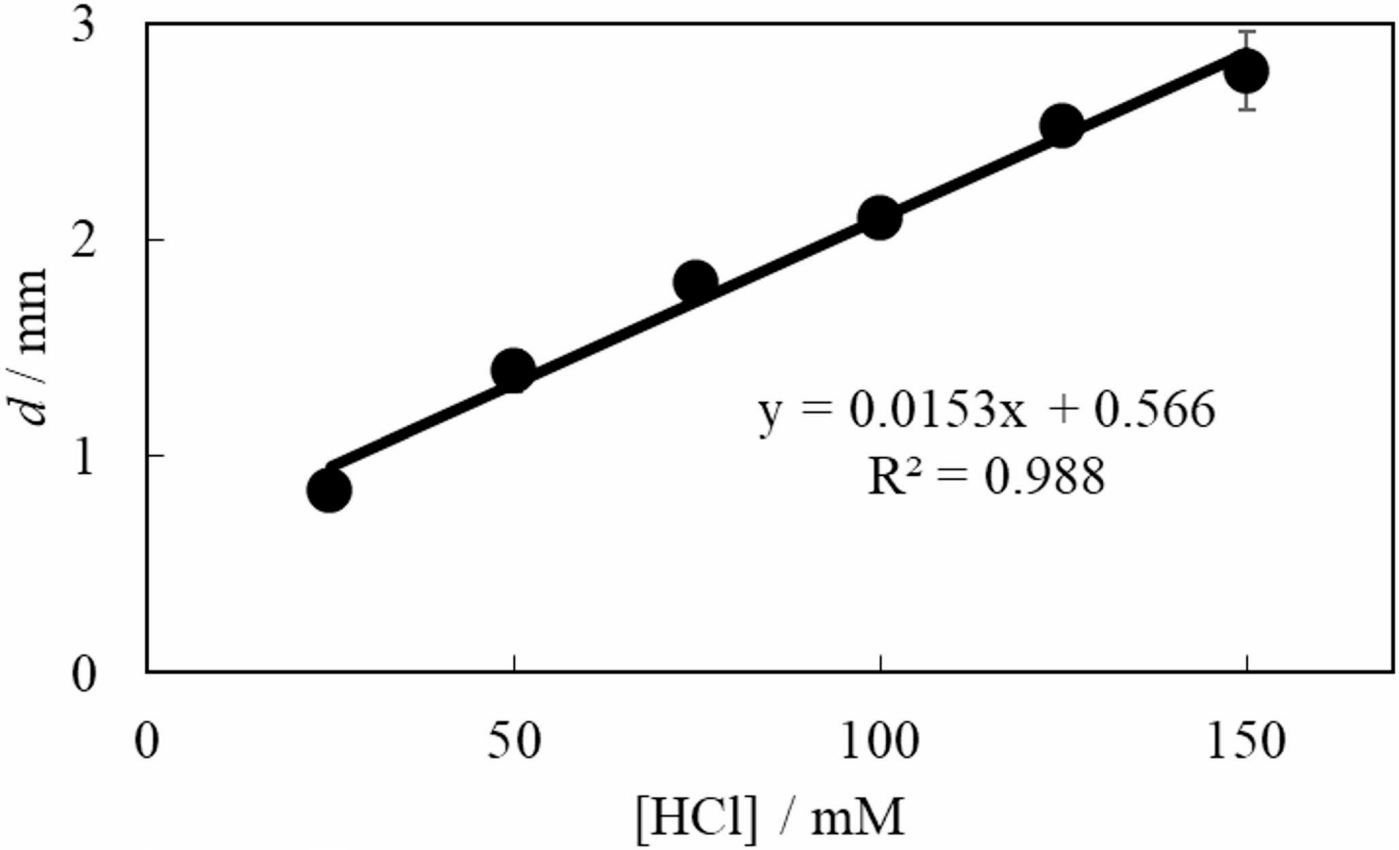
Calibration curve showing the relationship between distance *d* and acid concentration in the range of 25–150 mM for strong acid–strong base titration using a portable microfluidic device. (*n* = 3)

The extracted red channel image is shown in Fig. 6B, and the corresponding red intensity profiles are shown in Fig. 6C. With increasing acid concentration, the red intensity profile gradually changes from a steep to a more gradual shape. Based on these profiles, the position of the equivalence point within the channel was identified, and distance *d* was calculated. The resulting relationship between *d* and acid concentration is shown in Fig. 7. The calculated *d* values increase monotonically with increasing acid concentration, displaying a linear relationship over the investigated concentration range.

This behavior is consistent with the results previously obtained by microscopic observations and numerical simulations, confirming that quantitative acid concentration analysis can be achieved under smartphone-based imaging conditions using an appropriate device design.

### Application of the portable titration device to real sample analysis

The applicability and quantitative performance of the developed portable titration device were evaluated using real samples. Hot-spring water collected from the Tamagawa Hot Spring, which we reported on in the previous section, was subjected to acid–base titration.

**Fig. 8:**
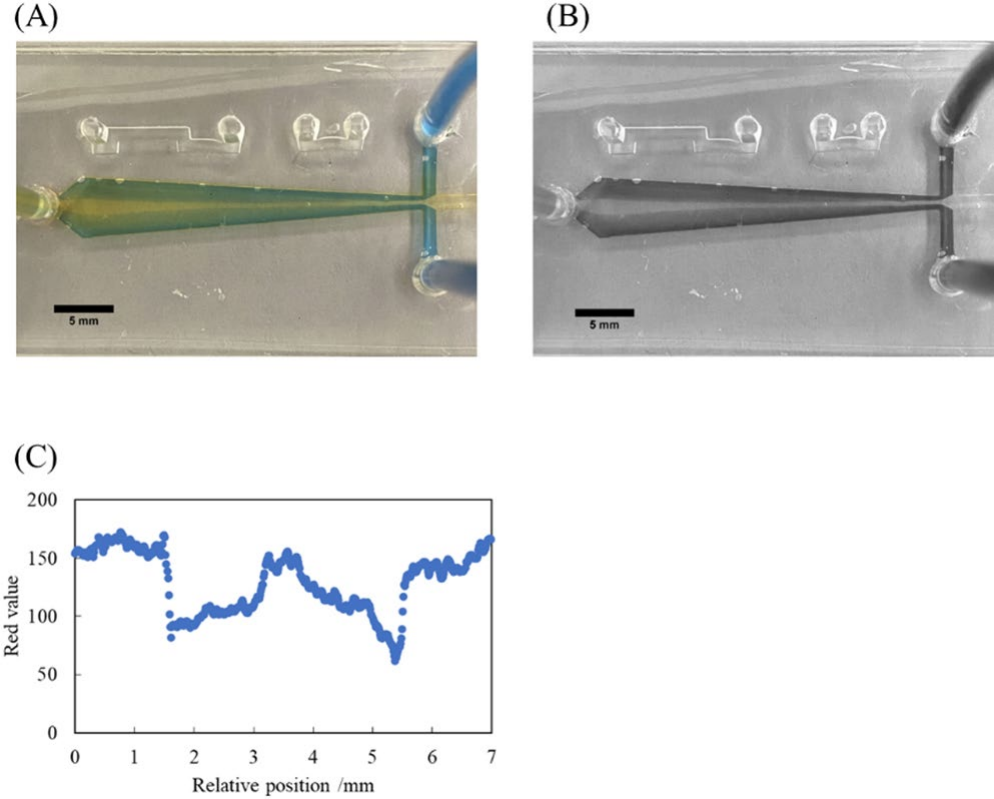
Representative images acquired during titration of a real sample using the portable titration device with a smartphone camera. **A** Original color image. **B** Image of extracted red channel. **C** Red value profile extracted from Fig. 8(B) along the direction perpendicular to flow

Representative images acquired during titration are shown in Fig. 8A. The extracted red channel image and corresponding red intensity profile are shown in Figs. 8B and C, respectively. Analysis of the red intensity profile yielded a distance *d* of 2.71 mm for the hot spring sample.

The calibration curve shown in Fig. 7 was obtained by linear least-squares fitting and can be expressed as:

1 $$d = \, 0.0{153}\left[ {{\mathrm{H}}^{ + } } \right] \, + \, 0.{566}$$

Substituting *d* = 2.71 mm into Eq. (1), the acid concentration of the hot-spring sample was determined to be 140 ± 3 mM.

To assess the accuracy of the portable titration device, the results were statistically compared with those obtained by conventional volumetric titration using the *t*-test. The results with the convetional volumetric titration was 141.6 ± 0.3 mM. The calculated *t* value was − 0.605, which is significantly smaller than the critical value of |*t*|= 4.302 at the 95% confidence level, indicating that there is no statistically significant difference between the two methods. In the previous chapter, titration performed under microscopic observation yielded an acid concentration of 143 ± 3 mM for the same sample, as opposed to the 140 ± 3 mM determined by the present portable system. Although a small difference of several millimolars was observed, the results fall within experimental error. This minor discrepancy is attributed to systematic differences arising from the device geometry and optical detection conditions. Although we employed the hand-made smartphone holder with gray-colored plastic, the experiment should be conducted where the stray light is omitted. Overall, these results demonstrate that the portable microfluidic titration device maintains high analytical accuracy and is suitable for onsite quantitative analysis.

This methodology requires the sample volume of less than 0.5 mL. In addition, the total analytical time, including sample flow, image acquisition, and image analysis, is ca. 5 min. This indicates that the reduction of both sample volume and analytical time accelerates the application of the present methodology to onsite analysis.

## Conclusion

In this study, a portable microfluidic titration system employing a smartphone camera as a detector was developed for onsite quantitative analysis. By designing an expanding-channel microfluidic device, the molecular diffusion within the channel can be enhanced, resulting in improved visual detectability of the equivalence point under smartphone-based imaging conditions. The optimized device enabled the acid–base titration of a strong acid–strong base system using bromothymol blue as the indicator, and a clear linear relationship was obtained between the measured distance and acid concentration over the range of 25–150 mM.

The applicability of the proposed system to real sample analysis was demonstrated by determining the acid concentration in hot-spring water. The results agreed with those obtained from conventional volumetric titration, with no statistically significant difference at the 95% confidence level. These findings demonstrate that quantitative titration analysis can be achieved without the use of microscopes or specialized optical instruments. The proposed approach provides a simple and practical platform for portable microfluidic titration and has the potential for on-site analytical applications in environmental and related fields.

## Supplementary Information

Below is the link to the electronic supplementary material.

Supplementary file1 (DOCX 651 kb)

## Data Availability

The data are available on reasonable request.
