# Supplementary material for: Portable microfluidic titration using a cross-shaped microfluidic device and smartphone camera for on-site quantitative analysis
Source: Anal Sci. 2026 May 12;42(8):805–13. doi: 10.1007/s44211-026-00902-4 (PMC13400585; doi:10.1007/s44211-026-00902-4)
Supplement: Supplementary file 1 — Supplementary file1 (DOCX 651 kb) [file 44211_2026_902_MOESM1_ESM.docx]

Supplementary Information

Advancements in Instrumentations

**Portable Microfluidic Titration Using a Cross-Shaped Microfluidic Device and Smartphone Camera for On-Site Quantitative Analysis**

Daina Numao, Nobuo Uehara, Arinori Inagawa*

*School of Engineering, Utsunomiya University, 7-1-2, Yoto, Utsunomiya, Tochigi, 321-8585, Japan*

Corresponding to ainagawa@a.utsunomiya-u.ac.jp


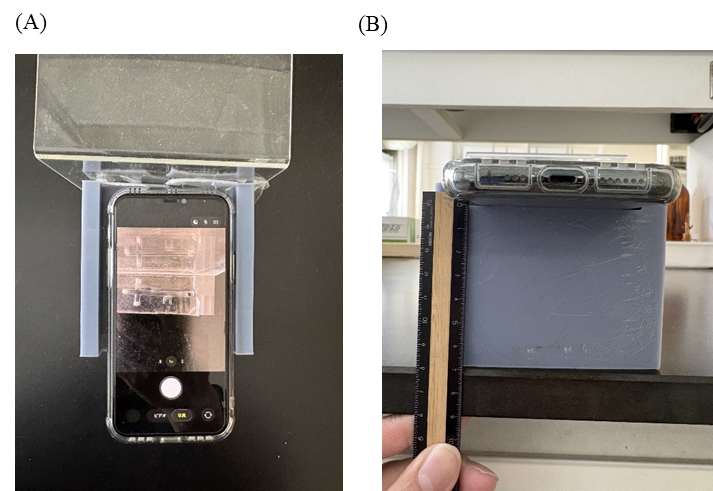


Fig. S1. A hand-made smartphone holder (A)top view (B) side view.
